# Psychostimulants influence oxidative stress and redox signatures: the role of DNA methylation

**DOI:** 10.1080/13510002.2022.2043224

**Published:** 2022-02-28

**Authors:** Vaishnavi Sundar, Tamizhselvi Ramasamy, Mayur Doke, Thangavel Samikkannu

**Affiliations:** a Department of Pharmaceutical Sciences, Irma Lerma Rangel College of Pharmacy, Texas A&M University, Kingsville, Texas, USA; b School of Biosciences and Technology, Vellore Institute of Technology, Vellore, India

**Keywords:** Psychostimulants, cocaine, methamphetamine, opioids, redox changes, epigenetics, methylation, neurodegeneration

## Abstract

**Objective:** Psychostimulant use induces oxidative stress and alters redox imbalance, influencing epigenetic signatures in the central nervous system (CNS). Among the various epigenetic changes, DNA methylation is directly linked to oxidative stress metabolism via critical redox intermediates such as NAD+, S-adenosylmethionine (SAM), and 2-oxoglutarate. Fluctuations in these intermediates directly influence epigenetic signatures, which leads to detectable alterations in gene expression and protein modification. This review focuses on recent advances in the impact of psychostimulant use on redox-imbalance-induced DNA methylation to develop novel epigenetics-based early interventions. **Methods:** This review is based on collective research data obtained from the PubMed, Science Direct, and Medline databases. The keywords used in the electronic search in these databases were redox, substance use disorder, psychostimulants, DNA methylation, and neurological diseases. **Results:** Instability in DNA methylation levels and redox expression effects are reported in various behavioral models stimulated by psychostimulants and opioids, indicating the widespread involvement of epigenetic changes in DNA methylation signatures in neurological disorders. **Discussion:** This review summarizes the need for more studies and experimental evaluations of DNA-methylation-based strategies that may help to understand the association between psychostimulant use and oxidative stress or redox-linked metabolic recalibration influencing neuronal impairments.

## Introduction

The United Nations Office on Drugs and Crime (UNODC) stated in the 2021 World Drug Report that 75 million people had used drugs worldwide, while over 36 million people had suffered from drug use disorders [1]. Illicit drugs are one of the major causes of neurobehavioral illness due to the irresistible urge to use these drugs, which are able to stimulate the central nervous system (CNS). Psychostimulants affect the cellular physiology of the brain through epigenetic modifications that result in neuronal impairment. Recent research shows that cellular redox status is an important predictor of epigenetic control that may have significant implications in human health and disease [2]. Furthermore, research has also shown that the oxidative stress induced by psychostimulant use plays a major role in neuropathology [3–6]. The changes in epigenetic modifications caused by psychostimulant use alter various cellular functions in the brain, including energy homeostasis, metabolic functions, mitochondrial integrity, inflammation and neuroplasticity [7,8]. Among these epigenetic modifications, DNA methylation predominantly regulates gene expression and protein modification in the brain during addiction [9]. DNA methylation changes are reported with drugs of abuse, such as cocaine, methamphetamine and opioids, which are all known to affect the CNS [10–13].

Neurological disorders are multifactorial disorders that are influenced by a combination of intrinsic and extrinsic factors, including genetic, epigenetic and environmental determinants. Moreover, genetic and environmental factors can particularly affect DNA methylation, resulting in cognitive dysfunction and behavioral disorders [14]. During DNA methylation, methyl groups are added to the 5′ positions of the pyrimidine rings of cytosine residues located in CpG dinucleotide islands, which impacts gene expression. This addition of methyl groups to cytosines in the DNA is regulated by the activity of DNA methyltransferases, induces synaptic plasticity in the hippocampal brain region and governs the neuroplasticity changes observed during psychostimulant use [15].

**Figure 1.**
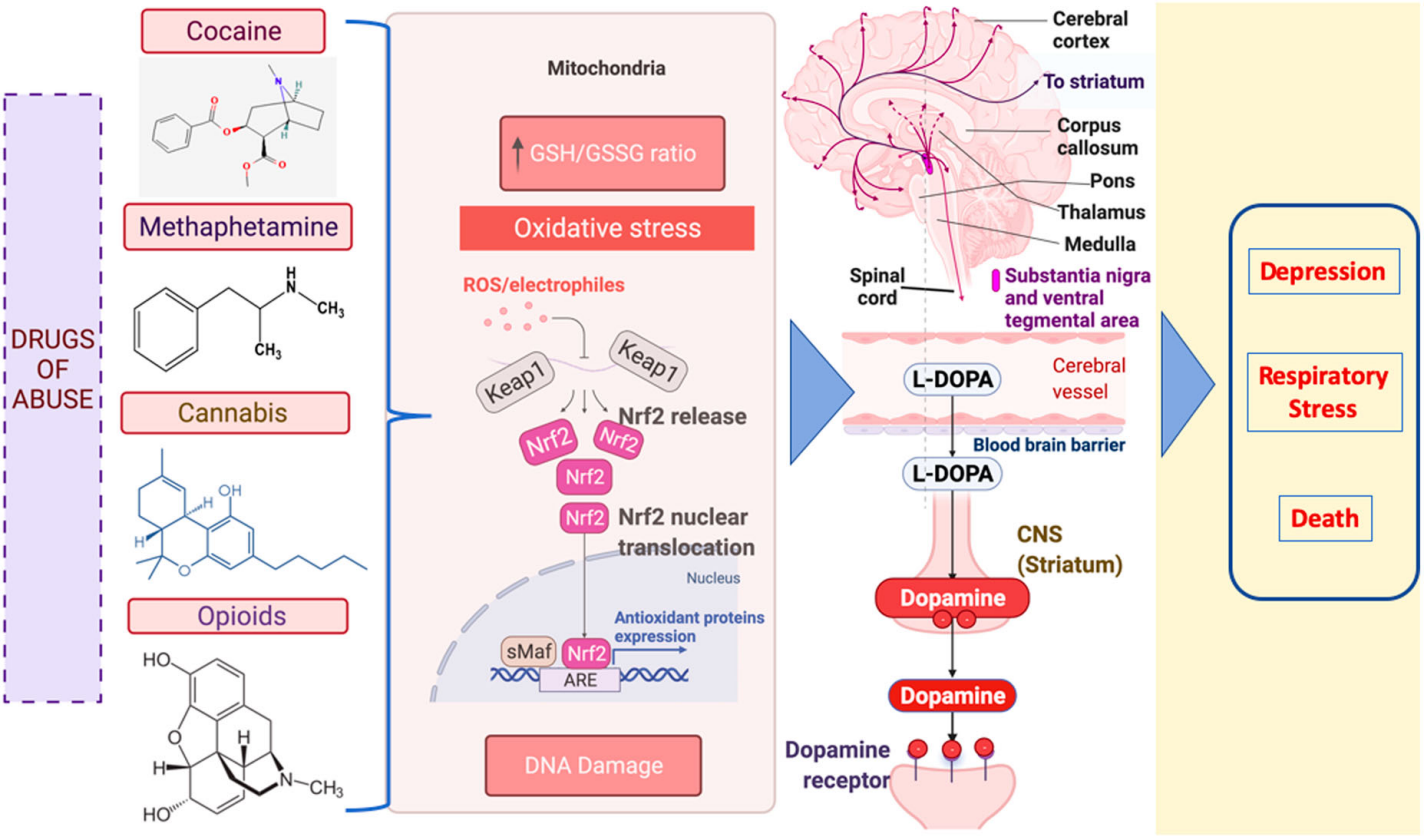
Dopamine release is upregulated by drug-induced redox imbalance in psychostimulant use. The illustration shows that the excess ROS produced due to the depletion of glutathione (GSH) in mitochondria promotes the oxidation of dopamine in neuronal cells. Consequently, dopamine autooxidation generates ROS again. The dopamine reaching its supramaximal levels in the brain due to psychostimulants generates oxidative stress attributed to the development of several neurodegenerative diseases. ARE -antioxidant response element; Nrf2 (nuclear erythroid 2-related factor 2); Keap1- Kelch-like ECH-associated protein 1;sMaf- Small Maf proteins.

Any psychostimulant-induced DNA methylation alterations notably harm the memory consolidation system in the hippocampus of the brain [16]. DNA methylation has also been implicated in neuropsychiatric diseases affecting cognitive functions by disrupting neurotransmitter activity [17]. However, a mechanistic link between neurotransmission and DNA methylation alterations induced by drugs of abuse has not been extensively explored. Moreover, drugs of abuse can also affect brain neurotransmission by stimulating neurons and transmitting signals in an abnormal pattern, disrupting the neural network [18,19]. For example, cocaine or methamphetamine triggers the release of pathologically increased amounts of neurotransmitters or interferes with the transporter mechanisms that aid in the replenishment of neurotransmitters [20]. Other drugs, such as opioids, cause disruptions in the brainstem, which controls heart rate, respiration, and the sleep cycle. Thus, an opioid overdose may result in depression, respiratory distress, and death [21]. The major neurotransmitter that is predominantly activated in the brain with drugs of abuse is dopamine, which is indirectly responsible for addiction by creating a desire for euphoria and repeated pleasurable activities in our body [20,22]. Interestingly, an increasing trend was found in the association of dopamine release with the redox imbalance induced by all types of drugs of abuse [3,23] (Figure 1).

**Figure 2.**
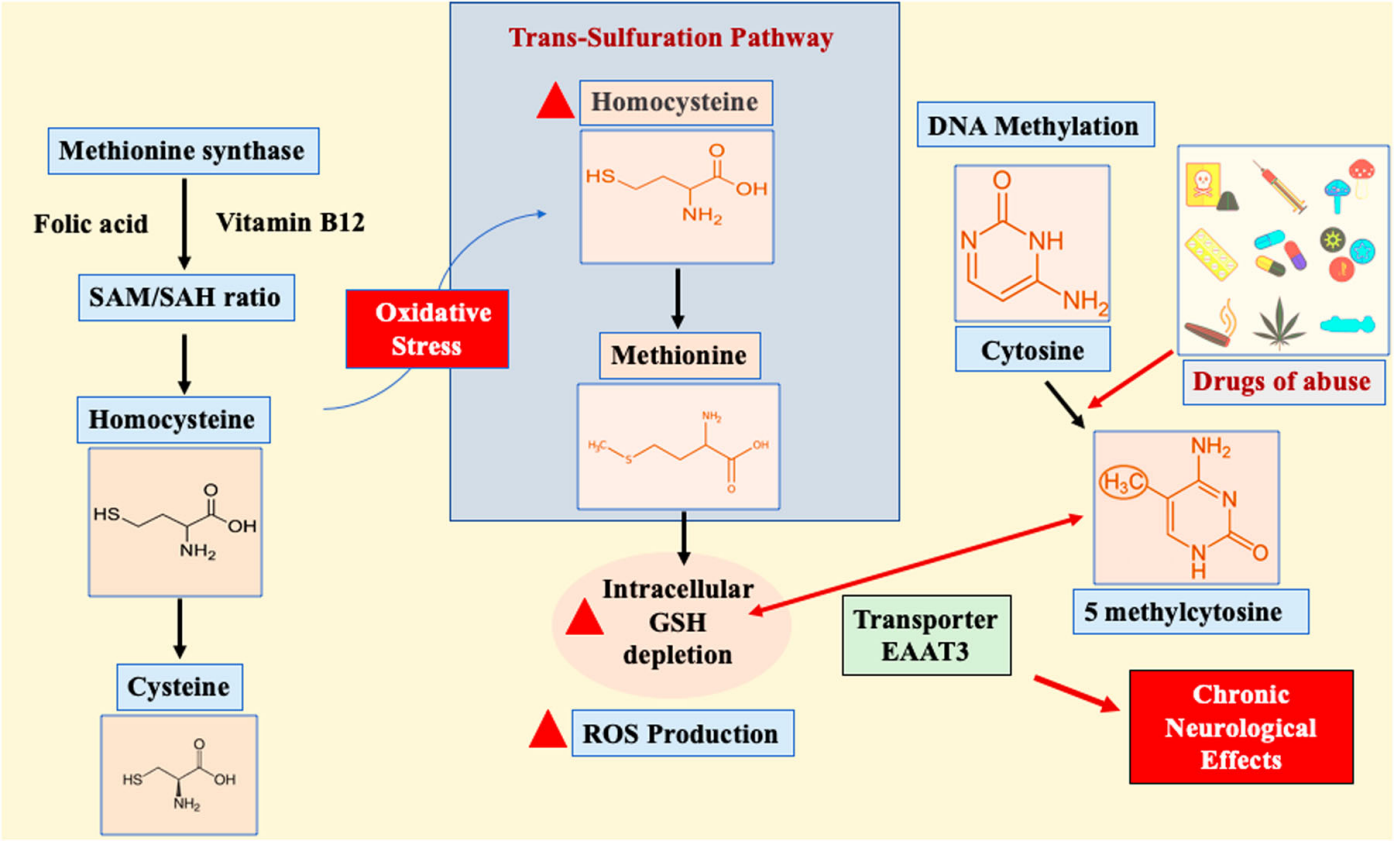
Excitatory amino acid transporter 3 (EAAT3)-mediated role of redox and DNA methylation in drugs of abuse. The S-adenosylmethionine (SAM)-to-S-adenosylhomocysteine (SAH) ratio is critically dependent on folic acid, and vitamin B12 controls methionine synthase (MS) activity. During oxidative stress, MS action is inhibited, resulting in the shift of the substrate homocysteine to the trans-sulfuration pathway to produce methionine. The increase in homocysteine levels depletes intracellular glutathione (GSH). However, in exceptional cases such as adult cortical neurons, cysteine is moved intracellularly by the excitatory amino acid transporter 3 (EAAT3), balancing cellular redox balance and DNA methylation reactions. The inhibition of EAAT3 activity causes neurodegeneration due to disturbances in redox and DNA methylation balance.

Recent studies have shown that altered cellular redox activity controls DNA methylation through the methionine synthase enzyme [24]. This enzyme is dependent on folic acid and vitamin B12 to control the ratio of the methyl group donor S-adenosylmethionine (SAM) to the methylation blocker S-adenosylhomocysteine (SAH)[25]. Methionine synthase inhibition by oxidative stress causes its substrate homocysteine to move to the trans-sulfuration pathway. Homocysteine is a sulfur-containing amino acid biologically derived from dietary methionine. However, homocysteine can be catabolized to cysteine or made available to form methionine via the trans-sulfuration pathway, specifically during oxidative conditions [3]. Hyperhomocysteinemia indirectly affects superoxide synthesis in the endothelium, leading to the depletion of intracellular glutathione (GSH) [26,27]. GSH depletion determines cellular redox status by donating electrons to oxidized proteins, and in some cases, the inhibition of the antioxidant enzyme glutathione peroxidase can be solely responsible for the increase in reactive oxygen species (ROS) causing oxidative stress [28,29]. However, adult cortical neurons have a very restricted trans-sulfuration pathway, and cysteine is moved intracellularly by excitatory amino acid transporter 3 (EAAT3) [30,31]. This transporter predominantly maintains cellular redox balance and actively controls DNA methylation reactions (Figure 2). Elevated plasma homocysteine levels are frequently linked to cerebrovascular and neurodegenerative diseases such as Alzheimer’s disease and neural tube defects [32,33]. The altered pharmacological mechanisms of the drugs of abuse need to be further evaluated in terms of their biochemical nature contributing to the development of chronic neurological effects, drug dependence, and abstinence. Moreover, drugs of abuse are known to influence redox status, and in the following sections, we will focus on their effects on GSH-induced redox imbalance and ROS production and how these alterations impact DNA methylation. The major purpose of this review is to highlight the involvement of redox imbalance and DNA methylation in drugs of abuse associated with pathological progression of neurological disorders. This review also sheds light on the association between DNA methylation and redox-based therapeutic strategies that attempt to combat the effects of so-called “drugs of abuse”.

### Redox mechanisms in substance abuse

As a metabolic byproduct of any cellular aerobic process, reactive oxygen species (ROS) influence a wide range of physiological events. Hence, their proper functionality is critical for redox homeostasis in cells. However, when the ROS levels exceed the cellular ability to neutralize these free radicals, oxidative stress occurs [29]. This oxidative stress may lead to cytotoxicity, including damage to macromolecules (nucleic acids, proteins, lipids), resulting in damage to cell organelles and eventually leading to cell death [34]. Notably, the neurotransmitter dopamine is the common mediator of mechanisms between structurally different drugs of abuse and oxidative stress, as it enhances drug-seeking behavior [35,36]. Additionally, dopamine contributes to brain ROS production and the maintenance of redox homeostasis in neuronal cells. In a normal physiological state, the enzyme monoamine oxidase in the mitochondria converts dopamine to 3,4-dihydroxyl phenylacetaldehyde by oxidative deamination, which produces hydrogen peroxide (H_2_O_2_), the primary form of ROS in the brain [37,38]. At pathological concentrations of dopamine, two consecutive single-electron oxidation reactions occur in neuronal cells, leading to the formation of dopamine o-quinone and two superoxide free radicals [39]. Furthermore, dopamine o-quinone conjugated with cysteine forms 5-S-glutathionyl-dopamine, and with glutathione, it forms 5-S-cysteinyl-dopamine in dopaminergic neurons and astrocytes [40,41]. Studies have shown that 5-S-cysteinyl dopamine is present in the cerebrospinal fluid and neuromelanin in humans [42]. However, 5-S-cysteinyl-dopamine is cytotoxic, as it triggers ROS production in human neuroblastoma (SH-SY5Y) cells [42,43]. Notably, H_2_O_2_ accumulation and ROS overproduction increase the autooxidation of dopamine in neuronal cells. On the other hand, this dopaminergic autooxidation promotes ROS production again. Therefore, dopamine reaching its supramaximal levels in the brain due to psychostimulants generates oxidative stress. The overproduction of mitochondrial ROS in CNS cells contributes to several neurodegenerative diseases with the abuse of illicit drugs. Furthermore, elevated ROS levels in mitochondria alter calcium-dependent protein kinase pathways, such as ERK, protein kinase C, protein kinase A, and CaM-kinases (CaMKII) [44–46], which are widely implicated in synaptic plasticity in drug addiction [47].

The structurally unrelated drugs of abuse have one thing in common—the “feeling of reward” caused by the increased dopamine levels. The reward system in the brain is affected by the changes in of ROS production. For example, cocaine treatment in rats increased the level of H_2_O_2_ and reduced GSH, which changes the redox status of the brain, stimulating the feeling of “reward” [48]. Findings from an animal model study support the notion that similar effects of chronic methamphetamine (METH) administration reduce glutathione levels in the nucleus accumbens and prefrontal cortex [49]. Recently, there have been more studies on the protective effect of ROS scavengers against METH abuse. Some of these include the phenyl-N-tert-butyl nitrone (PBN) action against METH-associated neurotoxicity due to ROS production [50], and 4-hydroxy-2,2,6,6-tetramethylpiperidine-1-oxyl (TEMPOL) specifically acts to prevent mitochondrial ROS imbalance in the rat brain [47,51]. However, there are some evidences available to show that superoxide dismutase (SOD) activity is also important in drugs abuse related neurotoxicity [52–54]. Supportively, in nontransgenic (non-Tg) mice, acute METH administration causes significant decrease in dopamine and dihydroxyphenylacetic acid whereas there were no significant decrease in dopamine in the SOD Tg mice indicating the potential importance of SOD in drugs abuse associated neurotoxicity [52,54]. Furthermore, METH-induced oxidative stress and neurotoxicity can be ameliorated by tert-butylhydroquinone (TBHQ), as it is influenced by the interplay between the nrf2-dependent antioxidative effects and the PI3K/AKT mechanism [55]. Furthermore, opioid-induced ROS production is regulated by peroxiredoxin 6 stimulation by dopamine receptors [56]. The consequential dopamine stimulation of abusive drugs results in oxidative stress and increases glutathione conjugation with free protein thiols mediated by S-glutathionylation, a redox-sensitive posttranslational modification. The antioxidant N-acetylcysteine reduces the accumulating glutamate levels in the nucleus accumbens by promoting the glutamate transporter (GLT-1) action that prevents the oxidative stress and neuroinflammation induced by drugs of abuse [3,43].

### Redox-based DNA methylation

Redox mechanisms are known to play a multitude of cellular physiological roles. Many recent studies have indicated that cellular redox levels are intrinsically associated with DNA methylation. Given this fact, experimental studies reveal that excessive ROS production is involved in changes in DNA methylation levels [57,58]. H_2_O_2_ transfers the proton at the C-5 cytosine position, promoting SAM production in the DNA methylation process [12]. In addition, ROS promote DNA methyltransferase 1 (DNMT) expression, which leads to site-specific hypermethylation in neurodegeneration [57,59]. Moreover, oxidative stress and redox proteins influence epigenetic changes directly or indirectly and regulate redox-dependent DNA methylation through brain iron levels, GSH metabolism, and the NAD+/NADH ratio [3]. Iron homeostasis in the brain is crucial, as it affects both receptor and transmitter activity in the brain. In general, a reduction in iron blocks dopamine transporter action, increasing synaptic dopamine concentrations by decreasing dopamine uptake. Moreover, the variation in the homeostatic iron regulator (HFE) protein gene increases the iron levels and promotes oxidative stress in the brain, thus increasing the risk for acquiring neurodegenerative disorders [60,61]. The HFE variant H67D mouse brain showed a significant reduction in 5 methylcytosine levels and reduced DNMT1 mRNA and protein expression. H67D mutation in HFE causes the deregulation of the redox-dependent methylation status of the brain by affecting the γ-aminobutyric acid (GABA) mechanism. The neurotransmitter GABA influences anxiety-related neurological disorders regulated by glutamate decarboxylase and GABA transaminase [62]. An *in vitro* study found that increased iron levels decrease DNMT activity and, hence, disturb the global DNA methylation status [63].

Moreover, excess iron affects hydrogen peroxide, depletes the antioxidant GSH, and promotes SAH production [60]. The increase in SAH hypomethylated DNA occurs through the inhibition of DNMT activity. The antioxidant-reduced GSH maintains cellular homeostasis by maintaining intracellular redox balance, and this GSH activity is based on NADPH generation via the pentose phosphate shunt pathway [64]. The occurrence of any changes in the cellular NAD levels that impact the promoter region of DNA methylation is known to alter brain-derived neurotrophic factor (BDNF) expression in CNS cells [65]. Furthermore, this DNA methylation at the promoter IV region silences BDNF by regulating methyl-CpG-binding protein (MeCP2), thus affecting transcriptional repression [66,67].

### Redox-associated DNA methylation in substance use disorder

It was previously believed that epigenetic modifications are imprinted during the development stages or inherited. However, a report has suggested that epigenetic mechanisms, similar to transcription, undergo continuous changes based on cues and the surrounding environment. Additionally, the study of epigenetic modifications may shed light on the ROS-related neurological abnormalities triggered by psychostimulants [68]. Drugs of abuse stimulate an increase in dopamine levels that potentially upregulates ROS production, resulting in undesired neurological effects. For instance, METH-treated rats show reduced GSH in the striatum and increased oxidative stress [69]. In addition, psychostimulants cause an increase in dopamine-dependent ROS production that affects MeCP2 function [70]. An increase in MeCP2 phosphorylation in the nucleus accumbens leads to increased behavioral sensitization [71]. In their *in vitro* and *in vivo* study, Kalda and colleagues showed that SAM levels are affected by DNMT3a downregulation, influencing cocaine-induced behavioral sensitization in mice [72]. Naturally, neurons predominantly rely on the EAAT3 transporter to obtain cysteine to synthesize GSH [58]. Notably, the enzyme methionine synthase regulates DNA methylation, which is significantly influenced by redox imbalance and GSH. Furthermore, drugs of abuse such as morphine show redox-dependent DNA methylation due to changes in cysteine levels due to EAAT3 activity [73]. In addition, L-b-threo-benzyl aspartate, which explicitly blocks EAAT3 activity, induces a decline in the GSH/GSSG and SAM/SAH ratios [74]. Furthermore, increased EAAT3 activity alters the cysteine residues on Kelch like ECH associated protein 1, which subsequently undergoes oxidation, causing Nrf2 disassociation, which affects cellular redox homeostasis [75]. The critical role played by EAAT3 in maintaining the redox balance in the human brain is well established, and EAAT3 may be considered a candidate target in the development of methylation-based therapeutics against psychostimulants.

### Redox-based epigenetics and therapeutics

The redox-based epigenetic mechanisms in psychostimulant use open a new avenue of pharmacological therapeutic targets against addiction. Moreover, epigenetic enzymes such as DNMTs depend on metabolic substrates and enzymes that can influence transcription and other pathophysiological events. Although many research studies focus on DNA-methylation-based therapeutics, a challenging aspect of this research is the targeting of specific brain regions. Therefore, more studies have used antioxidants such as N-acetylcysteine, PBN, and TEMPOL as potential targets to reduce the redox-dependent behavioral and molecular changes caused by drugs of abuse [28,76]. Apart from this, developing more reliable targeted drug delivery systems to deliver pharmacological agents to specific brain regions is an urgent need. However, the delay in developing effective treatment approaches to identify and deliver drugs to control drug abuse has resulted in an overwhelming economic burden.

Recently, IgG monoclonal antibody therapy against METH addiction has shown promise as a new approach against methamphetamine abuse. This approach uses METH-specific pharmacokinetic blockers to prevent METH from binding to the site of action [77]. In addition, multimodal therapeutics are crucial for controlling drug abuse based on the sustained activity of the medication. The ability to modify the pharmacokinetic parameters of the antibody against various drugs of abuse may prove to be more advantageous. Additionally, prolonged release of drugs to treat chronic drug abuse should be considered to tackle diverse drug abuse pathologies [78]. Nanomedicine has been shown to be an essential application of nanotechnology to address the challenge of delivering medications against drugs of abuse. The major challenge in delivering therapeutic agents into the CNS is the unpassable tight junctions of the blood–brain barrier (BBB). However, the advent of nanodelivery systems is paving the way for effective and versatile drug delivery to the CNS, as these systems permeate and transport drug molecules across the BBB through various molecular mechanisms. In addition, magnetic nanoparticle drug delivery has been introduced as a carrier of therapeutic agents against drugs of abuse [79]. A new iron oxide nanoformulation of a highly selective morphine blocker that prevents neuronal cell modulation during both HIV and morphine exposure has been developed [79].

Furthermore, advances in nanotheragnostic approaches have enhanced the sensitivity and selectivity of drug detection methods. For example, cocaine can influence the assembly of anti-cocaine aptamers (ACA-1 and ACA-2) by quenching the luminescence produced by gold nanoparticle-doped upconversion nanoparticles (UCNPs). In the presence of cocaine, the interactions between ACA-1 and ACA-2 are restored, and the UCNP luminescence quenched by the gold nanoparticles can be identified and roughly quantified by a smartphone camera [80]. Similar approaches can be used for other types of psychostimulants and recreational drugs. Therefore, integrating such nanomedicine advancements into current therapeutic interventions for drug abuse would provide a wide window for pharmacological breakthroughs for predictive and therapeutic approaches to tackle drugs of abuse associated with neurological disorders in the future.

## Conclusion

Drug abuse induces oxidative stress and affects redox status, influencing epigenetic changes in DNA methylation signatures and subsequently disrupting downstream gene transcription, resulting in neurological impairments. Further extensive research focusing on redox-based DNA methylation may open a Pandora’s box of novel pharmacological targets for metabolic interventions against drugs of abuse.

## Data Availability

Data sharing is not applicable to this article as no new data were created or analyzed in this study.
